# Robust Quantum State Tomography Method for Quantum Sensing

**DOI:** 10.3390/s22072669

**Published:** 2022-03-30

**Authors:** Ahmad Farooq, Uman Khalid, Junaid ur Rehman, Hyundong Shin

**Affiliations:** Department of Electronics and Information Convergence Engineering, Kyung Hee University, Yongin 17104, Korea; ahmadfarooq@khu.ac.kr (A.F.); umankhalid@khu.ac.kr (U.K.); junaid@khu.ac.kr (J.u.R.)

**Keywords:** quantum state tomography, depolarizing noise, quantum sensing, Heisenberg limit

## Abstract

Reliable and efficient reconstruction of pure quantum states under the processing of noisy measurement data is a vital tool in fundamental and applied quantum information sciences owing to communication, sensing, and computing. Specifically, the purity of such reconstructed quantum systems is crucial in surpassing the classical shot-noise limit and achieving the Heisenberg limit, regarding the achievable precision in quantum sensing. However, the noisy reconstruction of such resourceful sensing probes limits the quantum advantage in precise quantum sensing. For this, we formulate a pure quantum state reconstruction method through eigenvalue decomposition. We show that the proposed method is robust against the depolarizing noise; it remains unaffected under high strength white noise and achieves quantum state reconstruction accuracy similar to the noiseless case.

## 1. Introduction

High-dimensional pure entangled states are capitalized in applications, such as quantum communication, for higher information quantum capacity [1], provide security by enhancing the robustness against eavesdropping [2], the efficient distillation of resource states for implementing quantum computing algorithms [3], and optimal probe preparation for quantum sensing to ensure quadratic enhancements in precision scaling [4].

Quantum sensing is the science of making highly sensitive measurements of physical parameters under quantum entanglement [5]. Quantum sensor probes utilize the high intrinsic sensitivity of quantum systems towards minute perturbations while sensing any physical quantity. Moreover, pure entangled sensing probes benchmark classical sensors in terms of precision. However, the accurate reconstruction of such probes is extremely challenging due to the curse of inherent noise in the preparation process [6].

Experimentally, there have been numerous proposals on the preparation of high-dimensional probes on the physical architectures, such as superconducting circuits, color-center spins nuclear magnetic resonance, nitrogen vacancy center, Rydberg atoms, and trapped ions [1,7,8,9,10]. Here, we study the preparation of qudit probes with the highest possible accuracy under the depolarizing channel noise. This reconstruction problem of an unknown quantum state over finite ensembles *N* is known as quantum state tomography (QST). It utilizes statistical methodology to reconstruct the unknown quantum state over a finite number of registered data obtained from a set of measurement setups optimally chosen in advance. To fully reconstruct the mixed state, the set of measurement experiment setups should be informationally complete.

Maximum likelihood quantum state estimation (MLE) is a popular scheme used in quantum state tomography and is considered the standard. Sometimes, MLE yields a rank-deficient quantum state, which is a major drawback of this technique [11,12]. Bayesian mean estimation (BME) is proposed to tackle the rank-deficient problem imposed by the MLE [13,14,15,16]. Both MLE and BME become resource-intensive with the exponential growth of the *d*-dimensional qudit systems in the Hilbert space [17,18,19]. The computational cost of post-processing takes more time than the experiment itself. The linear regression estimation (LRE) technique exponentially reduces the computational cost of the post-processing after the measurement with a small amount of “accuracy sacrifice” [20,21]. The estimate through LRE may have negative eigenvalues and result in an invalid physical state due to the randomness of the measurement result. The technique is presented in [22] to revert the invalid density matrix to the physically valid quantum state. An adaptive pure state learning technique is presented in [23]. They have achieved very high accuracy for the higher-dimensional case. However, the algorithm achieves this feat by employing the changing basis at each iteration. Here, we provide a robust method using only a fixed measurement basis with high accuracy.

Reconstruction of the mixed quantum state requires $d^{2}$ measurement settings. It can further be decreased if the given unknown quantum state is pure [24]. The pure quantum state can only be realized with O$\left(d\right)$ measurement settings [25]. Recently, pure quantum state tomography has been demonstrated through three- and five-basis measurement settings [26,27]. The performance of the five-basis measurement settings is better than the three-basis measurement settings, as a function of the total number of unknown quantum state copies. In [28], the accuracy of estimation through the five-basis measurements further increases by modifying the reconstruction procedure. All of the described fixed-base algorithms are more prone to white noise. In a practical scenario, there is often white noise, which is a depolarizing noise in quantum systems. For the past few years, research has been directed towards the reconstruction of quantum state under depolarizing noise [28,29]. The authors in [28] devised an error-corrected modified five-basis (ECMFB) protocol in such a way that it could remove the white noise introduced during the tomography measurement process.

In this work, we will address the problem of accurate probe state reconstruction encountered in precise quantum sensing. Our solution revolves around the quantum state tomography accompanied by the eigenvalue decomposition to counter inherent depolarizing noise in the sensing probe preparation process. We analyze the quantum state reconstruction accuracy under the noisy data and prove that the pure quantum state extraction through our proposed technique is unaffected by the depolarizing noise. We also compare our proposal with ECMFB and reconstruct an optimal sensing probe, i.e., Bell state, under noise as a toy example.

## 2. Methods

A pure quantum state is defined by a unit norm vector and denoted as ket |ψ〉. The general representation of quantum state is density matrix, which is a mixture of pure state [30,31]

(1) $$\rho =\sum_{i}p_{i}|\psi_{i}\rangle \langle \psi_{i}|,$$

where $\sum_{i}p_{i}=1$. The experimental noisy view of the *d*-dimensional density matrix can be represented as

(2) $$\rho =\frac{1}{d}\sum_{i}m_{i}\sigma_{i},$$

where ${\left\{\sigma_{i}\right\}}_{i=1}^{d^{2}}$ is an orthonormal Hermitian operator and $m_{i}=tr\left(\rho \sigma_{i}\right)$ is the expectation value of $\sigma_{i}$ on the state ρ. These orthonormal Hermitian operator bases should be informationally complete. The generalized Gell-Mann operators are one kind of this, with $\sigma_{1}=I$. GGM operators are the generalized Pauli operators for higher-dimensional quantum systems [32]. These operators $\Lambda^{\left(m,n\right)}$ are (i) Hermitian $\Lambda^{\left(m,n\right)}=\Lambda^{(m,n)†}$, (ii) traceless tr$\left(\Lambda^{\left(m,n\right)}\right)=0,\;\forall \left(m,n\right)\neq \left(0,0\right)$, and (iii) obey the trace orthonormality relation tr$\left(\Lambda^{\left(u,v\right)}\Lambda^{\left(m,n\right)}\right)=\delta_{\left(u,v),(m,n\right)}$, where $\delta_{\left(u,v\right),{\left(m,n\right)}^{{}^{\prime }}}$ is the product of two Kronecker’s delta $\delta_{x,y}$ functions. On a *d*-dimensional Hilbert space, a total of $d^{2}-1$ GGM matrices are defined as follows [32].

1. $\frac{d\left(d-1\right)}{2}$ symmetric GGM
  $$\begin{array}{c} \Lambda_{s}^{\left(j,k\right)}=|j\rangle \langle k|+|k\rangle \langle j|,\;1\leq j<k\leq d, \end{array}$$
2. $\frac{d\left(d-1\right)}{2}$ antisymmetric GGM
  $$\begin{array}{c} \Lambda_{a}^{\left(j,k\right)}=-\dot{\iota }|j\rangle \langle k|+\dot{\iota }|k\rangle \langle j|,\;1\leq j<k\leq d, \end{array}$$
3. $\left(d-1\right)$ diagonal GGM
  $$\begin{array}{c} \Lambda^{\left(l,l\right)}=\sqrt{\frac{2}{l\left(l+1\right)}}\left(\sum_{j=1}^{l}|j\rangle \langle j|-l|l+1\rangle \langle l+1|\right),\;1\leq l\leq d-1. \end{array}$$

The probability of obtaining the outcome on a *j*th measurement basis is given by

(3) $$p_{j}=\langle j|\rho |j\rangle ,$$

where {|j〉} with j=1,2,⋯,d is the orthonormal bases of any GGM operator. In this paper, we will first calculate the expectation value of Bloch vectors for qudit with a generalized Gell-Mann basis. We reconstruct the density matrix using these expectation values. With the knowledge of an unknown rank-one pure state, we perform the spectral decomposition of the density matrix and select the eigenvector corresponding to the highest eigenvalue. This eigenvector is the desired unknown pure quantum state.

To reconstruct the quantum state ρ, we measure in GGM operators $\sigma_{i}=\Lambda^{\left(m,n\right)}$ and calculate all probabilities $p_{j}$ corresponding to the orthonormal basis of the operator. The expectation value of $\sigma_{i}$ operator in ρ is

(4) $$\begin{array}{cc} m_{i} & =tr\left(\rho \sigma_{i}\right)\\ \; & =tr\left(\rho \sum_{j}\mu_{j}|j\rangle \langle j|\right)\\ \; & =\sum_{j}\mu_{j}\langle j|\rho |j\rangle \\ \; & =\sum_{j}\mu_{j}p_{j}, \end{array}$$

where $\sigma_{i}=\sum_{j}\mu_{j}|j\rangle \langle j|$ is the eigen-decomposition of an operator $\sigma_{i}$. These expected values $m_{i}$ are used for quantum state tomography using the Equation (2). The resulting mixed state is in the form of a density matrix $\hat{\rho }$. To extract the pure state, we perform the spectral decomposition on the estimated density matrix and select the eigenvector $|\hat{\psi }\rangle$ corresponding to the highest eigenvalue. We find that the pure state tomography using this method shows distinguished results in the presence of depolarizing noise. The quantum state under depolarizing noise remains the same with probability $\left(1-\lambda \right)$ and with λ probability, the quantum state transforms into a maximally mixed quantum state

(5) $$N\left(\rho \right)=\left(1-\lambda \right)\rho +\frac{\lambda }{d}I,$$

where 0≤λ≤1. In the following Theorem, we will show the robustness of the state tomography algorithm under depolarizing noise.

**Theorem** **1.** *Using the eigenvector extraction from the standard state tomography algorithm, the estimated pure state passing through the depolarizing channel is robust under the noise strength 0≤λ<1.*

**Proof.**  Suppose we have the pure quantum state ρ=|ψ〉〈ψ|. The depolarizing noise acting on the quantum state transforms it into a mixed state

(6) $$N\left(\rho \right)=\left(1-\lambda \right)|\psi \rangle \langle \psi |+\frac{\lambda }{d}I.$$

First, we show that |ψ〉 is an eigenvector of $N\left(\rho \right)$

(7) $$\begin{array}{cc} N\left(\rho \right)|\psi \rangle & =\left(\left(1-\lambda \right)|\psi \rangle \langle \psi |+\frac{\lambda }{d}I\right)|\psi \rangle \\ \; & =\left(1-\lambda \right)|\psi \rangle +\frac{\lambda }{d}|\psi \rangle \\ \; & =\left(1-\lambda +\frac{\lambda }{d}\right)|\psi \rangle , \end{array}$$

which shows that $\left(1-\lambda +\frac{\lambda }{d}\right)$ and |ψ〉 are an eigenvalue and eigenvector of $N\left(\rho \right)$, respectively.

Next, we will show that the |ψ〉 is the eigenvector that belongs to the highest eigenvalue. By measuring the quantum state in an arbitrary unit vector |ϕ〉, we have

(8) $$\langle \phi |N\left(\rho \right)|\phi \rangle =\left(1-\lambda \right){\left|\langle \phi |\psi \rangle \right|}^{2}+\frac{\lambda }{d}.$$

From the above equation, we can see that the eigenvector corresponding to the highest eigenvalue only obtained when |ϕ〉=|ψ〉, and the corresponding eigenvalue is $\left(1-\lambda \right)+\frac{\lambda }{d}$, which completes our proof. □

We construct the estimated density matrix of the pure state under depolarizing noise with prior information of rank one of the unknown quantum state. The estimation through (2) under the depolarizing noise results in a mixed quantum state of $N\left(\hat{\rho }\right)$. If we select the highest eigenvector of $N\left(\hat{\rho }\right)$, it yields the actual estimated pure state $|\hat{\psi }\rangle$ as shown in Theorem 1. The Theorem suggests that our technique is robust against the depolarizing noise. We can further increase the performance of our algorithm. In any pure state, the tomography algorithm accuracy can be increased by combining the probability amplitude $|c_{k}|$ calculated by measuring in the computational basis with the estimate of complex phases $e^{\dot{\iota }\phi_{k}}$ using the solution of the QST pure state.

## 3. Results and Discussion

To compare the performance of our method, we used infidelity as a figure of merit. Infidelity is a distance measure between two arbitrary quantum states and is given as

(9) $$1-F\left(\rho ,\sigma \right)=1-{\left(tr\left(\sqrt{\sqrt{\rho }\sigma \sqrt{\rho }}\right)\right)}^{2}.$$

**Figure 1:**
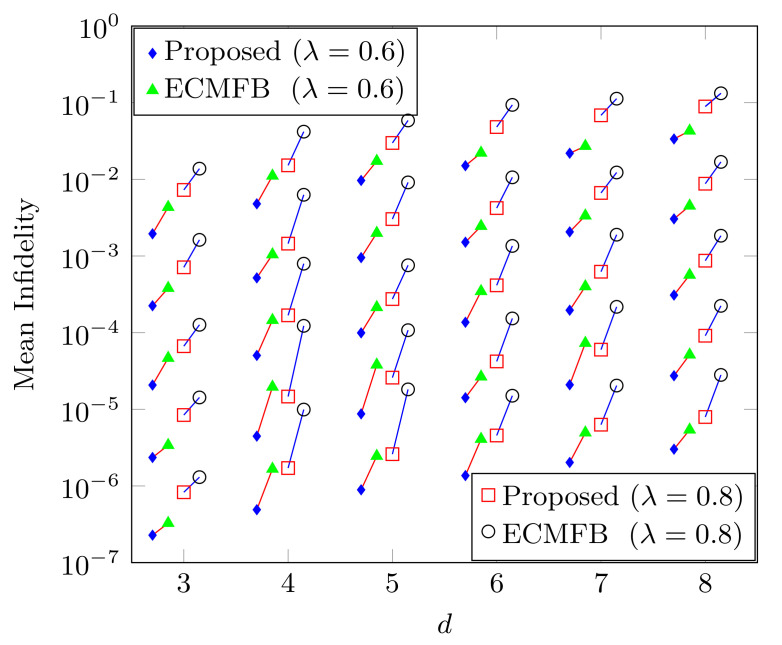
The mean infidelity of $10^{2}$ randomly generated pure quantum states according to the Haar measure with our method and ECMFB against *d*. The number of copies increases $10^{4}$ to $10^{8}$ as we move from the top to the bottom. The connection between two points represents the same number of copies with the same noise. The performance of our method is high in all dimensional cases.

We generate a $10^{2}$ pure quantum state randomly from the Haar measure. We plot the mean infidelity of these generated states obtained through our method and with the ECMFB method proposed in [28] against the dimension. The number of copies varies from $10^{4}$ to $10^{8}$ as we move from top to bottom. We can conclude from Figure 1 that our method is robust against the high depolarizing noise, and the performance is still higher than ECMFB.

**Figure 2:**
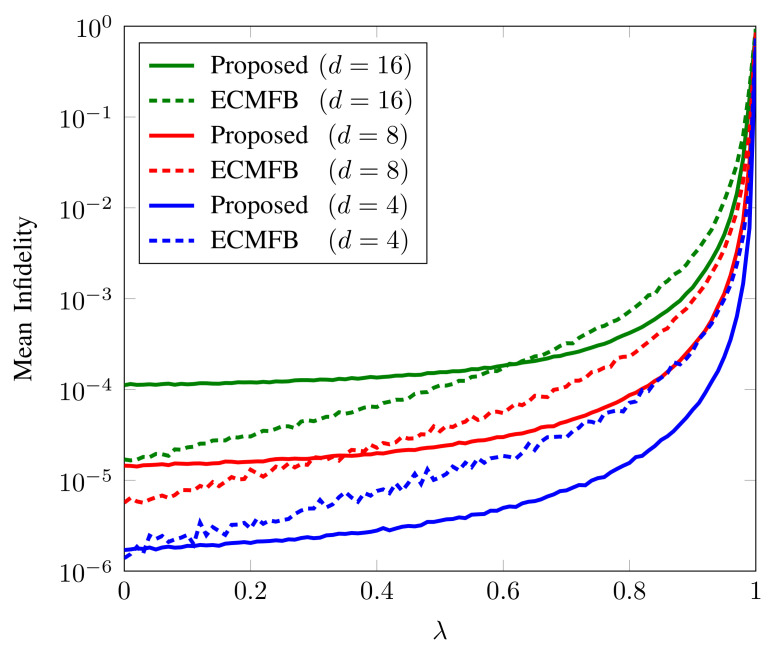
The mean infidelity of $10^{3}$ randomly generated pure quantum states according to the Haar measure of our algorithm and ECMFB against the λ with $N=10^{7}$. We can observe that our method shows more resilience towards the high noise strength.

To observe the accuracy with variable noise strength, we plot the mean infidelity of $10^{3}$ randomly generated states according to the Haar measure for the proposed scheme and ECMFB as a function of noise strength λ with $N=10^{7}$, where *N* is the total number of copies.. From Figure 2, we can see that, as the noise strength increases, our method remains robust against the noise. For higher-dimensional systems, the standard algorithm we are using utilizes $d^{2}$ measurement settings. In contrast, the algorithm in [28] only employs five bases for any dimensional system. Therefore, its accuracy is higher than our proposed scheme with low noise for high-dimensional systems. Although we have applied our method only to the standard state tomography algorithm, it can also be applied to any QST technique yielding the density matrix. We can use an algorithm with fewer measurement settings to obtain higher results for the qudits systems.

**Figure 3:**
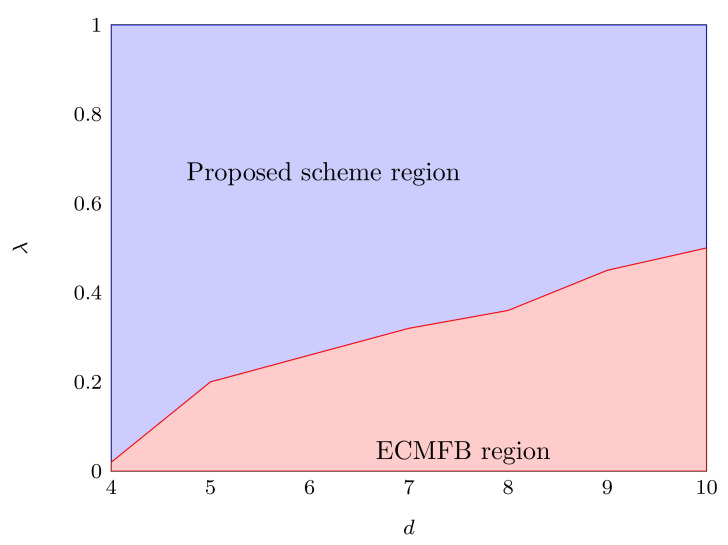
The interleaving position for $10^{3}$ was randomly generated from the Haar measure of the ECMFB and our algorithm with $N=10^{6}$. Our algorithm shows a high region for operating the QST problem under the depolarizing noise.

We have also plotted the interleaving position of the mean infidelity of $10^{3}$ randomly generated quantum states according to Haar measure between two algorithms. Figure 3 demonstrates that our algorithm has a more robust region as compared to the ECMFB for d=4,5,⋯,10.

To highlight the impact of our proposal, we employ Bell states, which are maximally entangled states in the two-qubit quantum systems. The maximum entanglement benchmarks Bell state-based quantum sensing schemes over classical sensing schemes wherein precision is limited by classical shot noise limit [33]. Therefore, reliable reconstruction of Bell state-based quantum sensing probes is a prerequisite in providing quadratic enhancements in achievable precision under noisy physical dynamics. To exemplify this, consider the following Bell state

(10) $$\begin{array}{c} |\psi \rangle =\frac{1}{\sqrt{2}}\left(|HH\rangle +|VV\rangle \right). \end{array}$$

**Figure 4:**
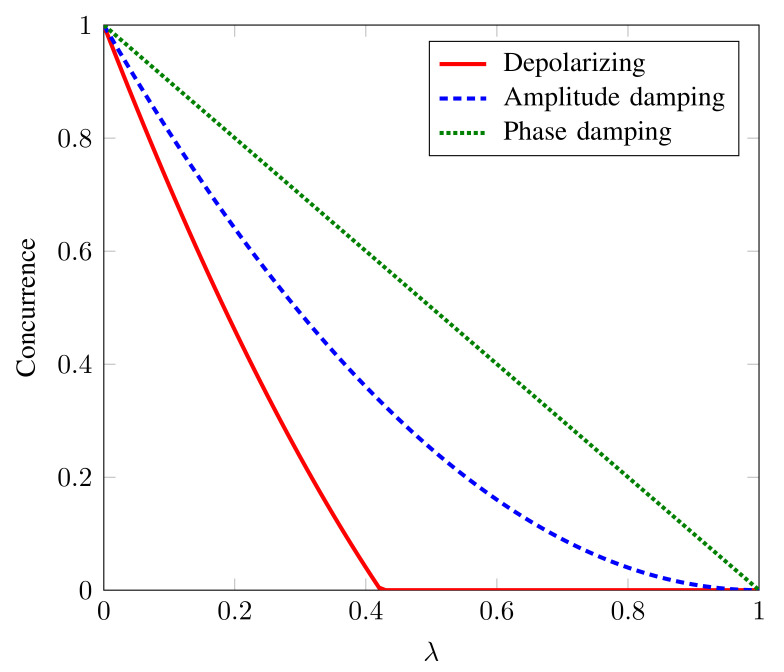
Entanglement (quantified by concurrence [34]) of the state (10) as a function of noise strength parameter λ.

**Figure 5:**
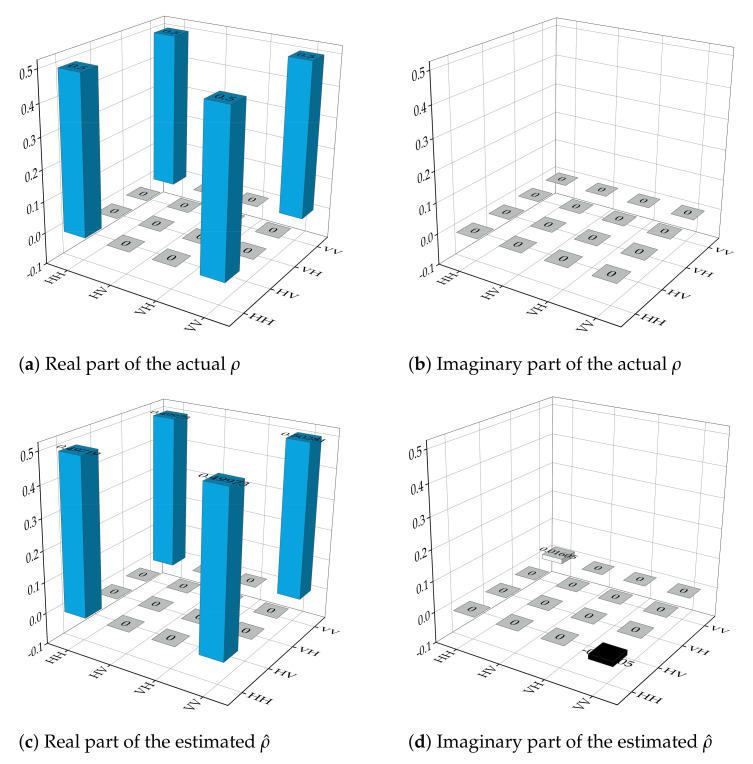
(**a**,**b**) represent real and imaginary parts of the actual Bell state. (**c**,**d**) represent the reconstructed Bell state under the depolarizing noise λ=0.8 with $10^{6}$ the number of copies.

We investigate the entanglement (quantified by concurrence) of the state (10) undergoing various noisy dynamics, namely, amplitude damping, phase damping, and depolarizing, as shown in Figure 4 [34]. It can be seen that entanglement vanishes for depolarizing noise strength λ>0.44. However, the Bell state is relatively robust against amplitude damping and phase damping noise. This sudden death of Bell state’s entanglement under depolarizing noise makes it unfeasible as quantum sensing probes since quantum advantage diminishes with entanglement sudden death. This signifies the dominance of the depolarizing noise against other types of decoherence with regard to optimal quantum sensing. Our algorithm, in particular, targets the reconstruction of quantum states in the presence of relatively dominant depolarizing noise. To visualize this, we perform the quantum state tomography of the given Bell state under the depolarizing noise strength of λ=0.8 and $N=10^{6}$ copies. In Figure 5, we plot the real and imaginary part of the reconstructed density matrix of the Bell state through our algorithm. Figure 5 shows that even with the noise strength being λ=0.8, we can accurately reconstruct the Bell state through our algorithm.

## 4. Conclusions

In this paper, we devised a scheme that provides higher quantum state reconstruction accuracy in the presence of a strong depolarizing noise. We also compared our robust scheme with the standard state-of-the-art ECMFB. We showed through numerical simulations that the proposed algorithm outperforms the existing algorithm under depolarizing noise. We can perform our scheme experimentally on a cloud quantum computer. In our QST problem, the measurement on the orthonormal basis is the only experimental part. On cloud superconducting IBM quantum computing, we have to provide an orthonormal basis that compiles the gates according to the given unitary, and makes an orthonormal unitary, to measure on an orthonormal set. In the future, we can employ alternate efficient state tomography algorithms that utilize fewer measurement settings to accurately reconstruct noise-robust sensing probes for optimal quantum sensing.

## Data Availability

The data generated from empirical results and the source code that support the findings of this study are available from the corresponding author upon reasonable request.

## Abbreviations

The following abbreviations are used in this manuscript:

QST	quantum state tomography
ECMFB	error-corrected modified five bases
GGM	generalized Gell-Mann
MLE	maximum likelihood estimation
LRE	linear regression estimation

## References

[1] Erhard M., Fickler R., Krenn M., Zeilinger A. (2018). Twisted Photons: New Quantum Perspectives in High Dimensions. Light. Sci. Appl..

[2] Bechmann-Pasquinucci H., Tittel W. (2000). Quantum Cryptography using Larger Alphabets. Phys. Rev. A.

[3] Campbell E.T., Anwar H., Browne D.E. (2012). Magic-State Distillation in All Prime Dimensions Using Quantum Reed-Muller Codes. Phys. Rev. X.

[4] Khalid U., ur Rehman J., Shin H. (2021). Metrologically Resourceful Multipartite Entanglement under Quantum Many-Body Effects. Quantum Sci. Technol..

[5] Degen C.L., Reinhard F., Cappellaro P. (2017). Quantum sensing. Rev. Mod. Phys..

[6] Torlai G., Mazzola G., Carrasquilla J., Troyer M., Melko R., Carleo G. (2018). Neural-network quantum state tomography. Nat. Phys..

[7] Neeley M., Ansmann M., Bialczak R.C., Hofheinz M., Lucero E., O’Connell A.D., Sank D., Wang H., Wenner J., Cleland A.N. (2009). Emulation of a Quantum Spin with a Superconducting Phase Qudit. Science.

[8] Soltamov V., Kasper C., Poshakinskiy A., Anisimov A., Mokhov E., Sperlich A., Tarasenko S., Baranov P., Astakhov G., Dyakonov V. (2019). Excitation and Coherent Control of Spin Qudit Modes in Silicon Carbide at Room Temperature. Nat. Commun..

[9] Klimov A.B., Guzmán R., Retamal J.C., Saavedra C. (2003). Qutrit Quantum Computer with Trapped Ions. Phys. Rev. A.

[10] Abobeih M., Randall J., Bradley C., Bartling H., Bakker M., Degen M., Markham M., Twitchen D., Taminiau T. (2019). Atomic-Scale Imaging of a 27-Nuclear-Spin Cluster using a Quantum Sensor. Nature.

[11] Banaszek K., D’Ariano G.M., Paris M.G.A., Sacchi M.F. (1999). Maximum-Likelihood Estimation of the Density Matrix. Phys. Rev. A.

[12] Opatrný T., Welsch D.G., Vogel W. (1997). Least-Squares Inversion for Density-Matrix Reconstruction. Phys. Rev. A.

[13] Blume-Kohout R. (2010). Optimal, Reliable Estimation of Quantum States. New J. Phys..

[14] Granade C., Ferrie C., Flammia S.T. (2017). Practical adaptive quantum tomography. New J. Phys..

[15] Blume-Kohout R. (2010). Hedged Maximum Likelihood Quantum State Estimation. Phys. Rev. Lett..

[16] Kazim S.M., Farooq A., ur Rehman J., Shin H. (2021). Adaptive Quantum State Tomography with Iterative Particle Filtering. Quantum Inf. Process..

[17] Shabani A., Kosut R.L., Mohseni M., Rabitz H., Broome M.A., Almeida M.P., Fedrizzi A., White A.G. (2011). Efficient Measurement of Quantum Dynamics via Compressive Sensing. Phys. Rev. Lett..

[18] Lloyd S., Mohseni M., Rebentrost P. (2014). Quantum Principal Component Analysis. Nat. Phys..

[19] Hou Z., Zhong H.S., Tian Y., Dong D., Qi B., Li L., Wang Y., Nori F., Xiang G.Y., Li C.F. (2016). Full Reconstruction of a 14-Qubit State within Four Hours. New J. Phys..

[20] Qi B., Hou Z., Li L., Dongi D., Xiang G., Guo G. (2013). Quantum State Tomography via Linear Regression Estimation. Sci. Rep..

[21] Qi B., Hou Z., Wang Y., Dong D., Zhong H.S., Li L., Xiang G.Y., Wiseman H.M., Li C.F., Guo G.C. (2017). Adaptive Quantum State Tomography via Linear Regression Estimation: Theory and Two-Qubit Experiment. npj Quantum Inf..

[22] Smolin J.A., Gambetta J.M., Smith G. (2012). Efficient Method for Computing the Maximum-Likelihood Quantum State from Measurements with Additive Gaussian Noise. Phys. Rev. Lett..

[23] Rambach M., Qaryan M., Kewming M., Ferrie C., White A.G., Romero J. (2021). Robust and Efficient High-Dimensional Quantum State Tomography. Phys. Rev. Lett..

[24] Steffens A., Riofrío C.A., McCutcheon W., Roth I., Bell B.A., McMillan A., Tame M.S., Rarity J.G., Eisert J. (2017). Experimentally Exploring Compressed Sensing Quantum Tomography. Quantum Sci. Technol..

[25] Kueng R., Rauhut H., Terstiege U. (2017). Low Rank Matrix Recovery from Rank One Measurements. Appl. Comput. Harmon. Anal..

[26] Goyeneche D., Nas G.C., Etcheverry S., Gómez E.S., Xavier G.B., Lima G., Delgado A. (2015). Five Measurement Bases Determine Pure Quantum States on Any Dimension. Phys. Rev. Lett..

[27] Zambrano L., Pereira L., Martínez D., Nas G.C., Lima G., Delgado A. (2020). Estimation of Pure States Using Three Measurement Bases. Phys. Rev. Appl..

[28] Zambrano L., Pereira L., Delgado A. (2019). Improved Estimation Accuracy of the 5-Bases-Based Tomographic Method. Phys. Rev. A.

[29] Torlai G., Melko R.G. (2018). Latent Space Purification via Neural Density Operators. Phys. Rev. Lett..

[30] Nielsen M.A., Chuang I. (2002). Quantum Computation and Quantum Information.

[31] Wilde M.M. (2013). From Classical to Quantum Shannon Theory.

[32] Bertlmann R.A., Krammer P. (2008). Bloch Vectors for Qudits. J. Phys. A Math. Theor..

[33] Khalid U., Jeong Y., Shin H. (2018). Measurement-based quantum correlation in mixed-state quantum metrology. Quantum Inf. Process..

[34] Im D.G., Kim Y.H. (2022). Decoherence-Induced Sudden Death of Entanglement and Bell Nonlocality. Photonics.

